# Changes in carbon dioxide production and oxygen uptake evaluated using indirect calorimetry in mechanically ventilated patients with sepsis

**DOI:** 10.1186/s13054-021-03830-z

**Published:** 2021-12-04

**Authors:** Ichiro Hirayama, Toshifumi Asada, Miyuki Yamamoto, Naoki Hayase, Takahiro Hiruma, Kent Doi

**Affiliations:** Department of Emergency and Critical Care Medicine, The University of Tokyo, 7-3-1 Hongo, Bunkyo-ku, Tokyo, 113-0033 Japan

**Keywords:** Carbon dioxide production, Oxygen extraction, Indirect calorimetry, Sepsis, Lactate

## Abstract

**Background:**

Several clinical guidelines recommend monitoring blood lactate levels and central venous oxygen saturation for hemodynamic management of patients with sepsis. We hypothesized that carbon dioxide production (VCO_2_) and oxygen extraction (VO_2_) evaluated using indirect calorimetry (IC) might provide additional information to understand the dynamic metabolic changes in sepsis.

**Methods:**

Adult patients with sepsis who required mechanical ventilation in the intensive care unit (ICU) of our hospital between September 2019 and March 2020 were prospectively enrolled. Sepsis was diagnosed according to Sepsis-3. Continuous measurement of VCO_2_ and VO_2_ using IC for 2 h was conducted within 24 h after tracheal intubation, and the changes in VCO_2_ and VO_2_ over 2 h were calculated as the slopes by linear regression analysis. Furthermore, temporal lactate changes were evaluated. The primary outcome was 28-day survival.

**Results:**

Thirty-four patients with sepsis were enrolled, 26 of whom survived 76%. Significant differences in the slope of VCO_2_ (− 1.412 vs. − 0.446) (*p* = 0.012) and VO_2_ (− 2.098 vs. − 0.851) (*p* = 0.023) changes were observed between non-survivors and survivors. Of note, all eight non-survivors and 17 of the 26 survivors showed negative slopes of VCO_2_ and VO_2_ changes. For these patients, 17 survivors had a median lactate of − 2.4% changes per hour (%/h), whereas non-survivors had a median lactate of 2.6%/hr (*p* = 0.023).

**Conclusions:**

The non-survivors in this study showed temporal decreases in both VCO_2_ and VO_2_ along with lactate elevation. Monitoring the temporal changes in VCO_2_ and VO_2_ along with blood lactate levels may be useful in predicting the prognosis of sepsis.

**Supplementary Information:**

The online version contains supplementary material available at 10.1186/s13054-021-03830-z.

## Introduction

Sepsis is a leading cause of death in intensive care units (ICUs) [1, 2]. Hemodynamic management in patients with sepsis is important for providing a sufficient amount of oxygen to the organs and preventing the development of multiple organ dysfunction. Several therapeutic strategies for sepsis, such as early goal-directed therapy [3] and Hour-1 Bundle [4], include hemodynamic management. Measuring blood lactate level and its temporal changes (lactate clearance) and monitoring central venous oxygen saturation (S_CV_O_2_) improved the outcomes of patients with sepsis [5–8]. However, these indicators have some limitations. Some studies have reported that interventions with S_CV_O_2_ monitoring failed to show better outcomes [9]. Blood lactate levels will be affected by liver dysfunction [10]. Therefore, some other monitoring indicators are needed for clinically managing sepsis.

Sepsis is characterized by altered cellular metabolism and impaired oxygen usage despite adequate oxygen delivery (DO_2_) [11]. Recently, mitochondrial impairment termed “cytopathic hypoxia” has been recognized as the mechanism of organ dysfunctions in sepsis [12]. Under the decrease in oxygen extraction, the mitochondria cannot generate energy via oxidative phosphorylation, and energy metabolism will become dependent on anaerobic glycolysis under hypoxic state [13]. Monitoring carbon dioxide production (VCO_2_) and oxygen extraction (VO_2_) is expected to help detect the progression of sepsis exacerbation, especially impaired oxygen usage in the mitochondria. Indirect calorimetry (IC) can simultaneously and noninvasively measure VCO_2_ and VO_2_. It has already been used in ICUs for measuring energy expenditure and oxygen consumption [14, 15]. IC will provide information not only for estimated nutritional requirements but also for tissue metabolism [16].

This study was designed to explore the possible role of IC in monitoring cellular oxygen metabolism in patients with sepsis. We measured the temporal changes in VCO_2_ and VO_2_ in mechanically ventilated patients with sepsis and evaluated whether these parameters are associated with 28-day survival.

## Method

### Study design and participants

**Fig. 1:**
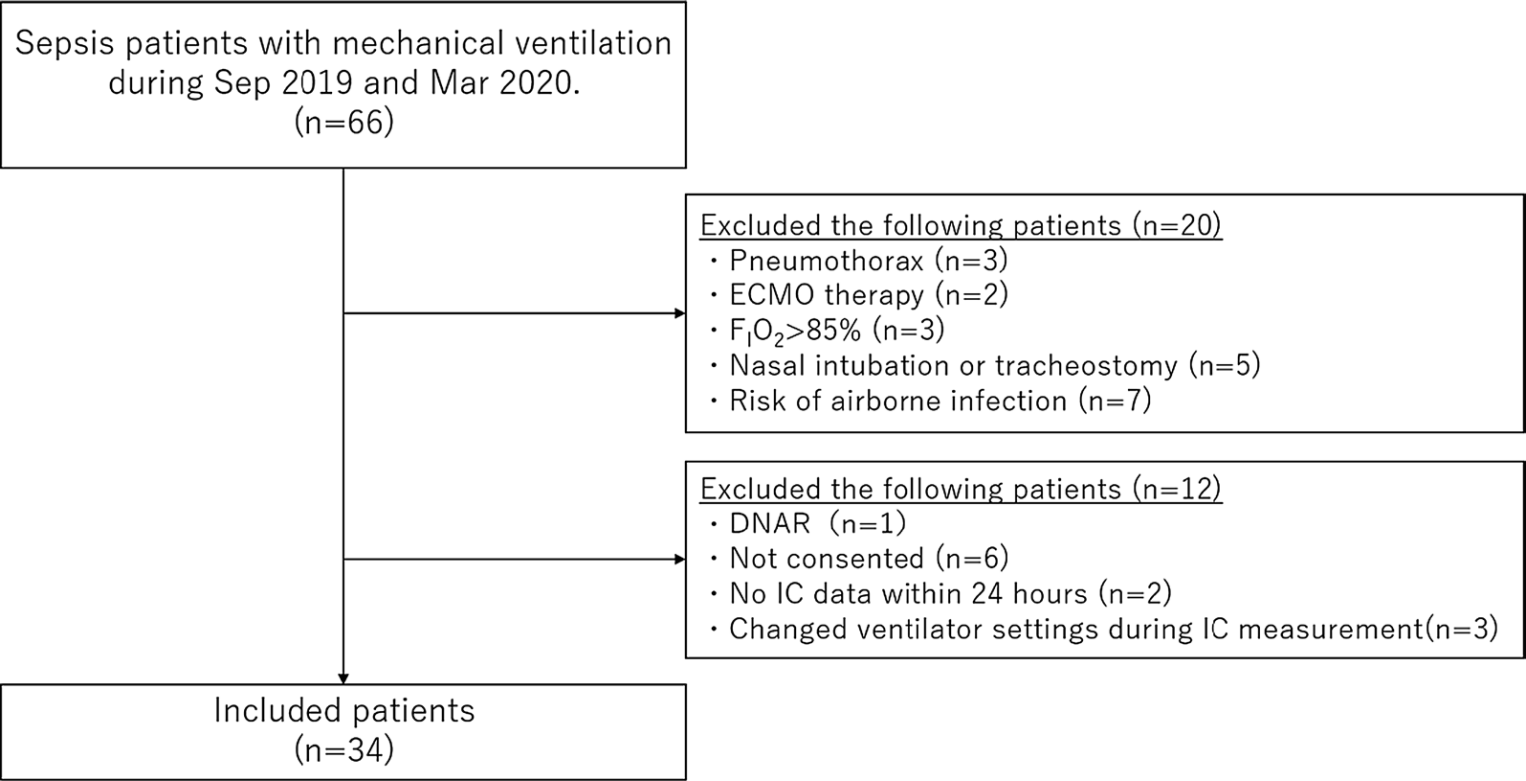
Flowchart of the study population. DNAR, do not attempt resuscitation; ECMO, extracorporeal membrane oxygenation; F_I_O_2_, fraction of inspired oxygen; IC, indirect calorimetry

This study was a single-center prospective observational study, which has been registered on the UMIN Clinical Trials Registry (registry number: UMIN 000045966). Adult patients (> 18 years old), who were diagnosed with sepsis and orally intubated in the ICU of the University of Tokyo Hospital between September 2019 and March 2020, were included. Sepsis was diagnosed according to Sepsis-3 [17]. We excluded the following patients because of the inaccuracy of IC measurement: patients complicated with pneumothorax, those treated with extracorporeal membrane oxygenation (ECMO) therapy, those ventilated with more than 85% fraction of inspired oxygen (F_I_O_2_), those with changes in ventilator settings including F_I_O_2_ during IC measurement, those intubated from nasal or tracheostomy, and those isolated for high risk of airborne infection. Moreover, we excluded patients who declared do not attempt resuscitation and those without informed consent. During the study period, 66 patients with sepsis were mechanically ventilated in our ICU, and 34 patients were finally enrolled in this study (Fig. 1). IC measurement was initiated within 24 h after oral tracheal intubation. VCO_2_ and VO_2_ values were measured for 2 h continuously.

This study was conducted according to the amended Declaration of Helsinki, and the Institutional Review Board of the University of Tokyo approved this study (2018094NI). Informed consent was obtained from all participants or their legal representatives.

### Indirect calorimetry

For IC, CCM Express (MGC Diagnostics, Saint Paul, Minnesota) [18] was used. Warm-up and calibration were conducted according to the specifications of the manufacturer. IC measures the difference between inspiratory and expiratory VCO_2_ and VO_2_ using the breath-by-breath analysis method. It uses a pneumotach flowmeter connected near the endotracheal tube. Inspiratory and expiratory gases were collected through a sampling line connected to this flowmeter. VCO_2_ is measured using an infrared analyzer, while VO_2_ is measured using a galvanic fuel cell. Patient ventilation is measured at the endotracheal tube. Therefore, considering any bias flow provided by the ventilator is not needed [19, 20].

### Data collection

The following patient characteristics and clinical data were collected from the medical records: age, sex, past medical history, height, weight, catecholamine use, continuous renal replacement therapy use, induction medication, sedation, Richmond Agitation-Sedation Scale　(RASS), source of infection, thiamine administration, and positive results of the culture. Blood gas analysis, including blood lactate levels, was performed during intubation and after IC measurement. Furthermore, ventilator settings and vital signs during IC measurement were obtained. The Acute Physiology and Chronic Health Evaluation (APACHE) II score, Sequential Organ Failure Assessment (SOFA) score, and catecholamine index were calculated.

VCO_2_, VO_2_, and respiratory quotient (RQ) were measured using IC. Before the analysis, VCO_2_, VO_2_, and RQ data obtained using IC were modified to minimize the possible artifacts in the following process (Additional file 1: Fig. S1). First, the values considered out of the physiological range (VCO_2_ < 70 mL/min or > 800 mL/min; VO_2_ < 100 mL/min or > 1000 mL/min; and RQ < 0.67 or > 1.3) were excluded [21–23]. Second, the average values every 5 min (24 points for 2 h) in each of VCO_2_, VO_2_, and RQ were obtained. The outlier values outside the mean ± 2 standard deviation of 24 points and points before and after the outlier values were excluded. Finally, a linear regression line was obtained from the remaining points, and each slope was defined as VCO_2_, VO_2_ and RQ slopes.

For temporal changes in lactate levels, the percentage of change was measured hourly (%/hr); the percentage of changes in blood lactate level was obtained by dividing the hours from two time points (during intubation and the end of IC measurement).

### Outcomes

The primary outcome was 28-day survival, and its association with VCO_2_ and VO_2_ slopes was evaluated. We further evaluated the additional information provided by VCO_2_ and VO_2_ slopes along with lactate temporal changes.

### Statistical analysis

Continuous variables were presented as median (interquartile range), and categorical variables were presented as percentages. Categorical data were compared using the chi-square test or Fisher’s exact test as appropriate. Multivariate logistic regression analysis was performed to examine the associations of VCO_2_ and VO_2_ slopes with 28-day mortality adjusted from the predefined confounding factors of APACHE II score and lactate temporal changes. Predictive performance of each parameter for 28-day mortality was evaluated by receiver operating characteristic (ROC) analysis, and the cutoff values were determined with Youden’s index. All statistical analyses were performed using JMP Pro (version 15.1.0; SAS Institute Inc., Cary, NC, US). Two-tailed *p* values of less than 0.05 were used to denote statistical significance for all tests.

## Results

### Patient characteristics

Among the 34 enrolled patients, 26 survived and eight died within 28 days after ICU admission. The characteristics and clinical parameters are shown in Table 1. Age and APACHE II scores in non-survivors were significantly higher than those in survivors. Moreover, a significant difference in lactate temporal changes was observed between non-survivors and survivors. No significant differences in other characteristics and parameters were observed between these groups. Average values of VCO_2_, VO_2_, RQ, and REE during 2 h IC measurement in each patient are shown in Additional file 2: Table S1.

**Table 1:** Characteristics and outcomes of mechanically ventilated patients with sepsis

	Total		Survivors		Non-survivors		*p* value
	N = 34		N = 26		N = 8		
Age, y	73	(51.8–81.0)	65.5	(46.0–78.5)	81	(72.8–84.0)	0.023
Male sex	24	(70.6)	19	(73.1)	5	(62.5)	0.666
BMI, kg/m^2^	20.9	(17.1–25.2)	21.5	(17.1–25.8)	19.7	(15.7–23.7)	0.372
*Clinical severity*							
APACHE II score	17	(12.0–22.0)	15	(11.0–21.0)	21.5	(17.0–24.5)	0.046
SOFA score	9	(5.8–12.0)	7.5	(5.0–11.3)	11	(8.3–13.0)	0.154
*Catecholamine use*							
Noradrenalin	23	(67.6)	17	(65.4)	6	(75)	1
Dopamine	1	(2.9)	1	(3.8)	0	(0)	1
Dobutamine	3	(8.8)	3	(11.5)	0	(0)	1
Vasopressin	9	(26.5)	6	(23.1)	3	(37.5)	0.649
Catecholamine index	19	(0.0–30.0)	15	(0.0–40.0)	27.5	(5.0–30.0)	0.605
*Induction medication*							
Fentanyl	33	(97.1)	26	(100)	7	(87.5)	0.235
Propofol	22	(64.7)	19	(73.1)	3	(37.5)	0.098
Midazolam	12	(35.3)	8	(30.8)	4	(50)	0.41
Sedation							
Fentanyl	32	(94.1)	26	(100)	6	(75)	0.05
Propofol	26	(76.5)	20	(76.9)	6	(75)	1
Midazolam	1	(2.9)	1	(3.8)	0	(0)	1
Dexmedetomidine	9	(26.5)	8	(30.8)	1	(12.5)	0.403
RASS (during IC measurement)	− 4	(− 4 to − 3)	− 4	(− 4 to − 3)	− 4	(− 4 to − 3)	0.946
CRRT	2	(5.8)	1	(3.8)	1	(12.5)	0.421
*Past history*							
Hypertension	14	(41.2)	11	(42.3)	3	(37.5)	1
Diabetes mellitus	13	(38.2)	11	(42.3)	2	(25)	0.444
Ischemic heart disease	3	(8.8)	2	(7.7)	1	(12.5)	1
Stroke	4	(11.8)	4	(15.4)	0	(0)	0.552
COPD	6	(17.6)	4	(15.4)	2	(25)	0.609
Chronic liver disease	4	(11.8)	4	(15.4)	0	(0)	0.552
CKD	5	(14.7)	3	(11.5)	2	(25)	0.57
Cancer	10	(29.4)	8	(30.8)	2	(25)	1
*Source of infection*							
Respiratory	22	(64.7)	16	(61.5)	6	(75)	0.681
Urinary	3	(8.8)	2	(7.7)	1	(12.5)	1
Biliary	4	(11.8)	3	(11.5)	1	(12.5)	1
Other	5	(14.7)	5	(19.2)	0	(0)	0.309
Positive culture	10	(29.4)	8	(30.8)	2	(25)	1
*Ventilator*							
Assist/control mode	21	(61.8)	17	(65.4)	4	(50)	0.68
F_I_O_2_, %	40	(35.0–60.0)	40	(35.0–52.5)	40	(30.0–67.5)	0.95
PEEP, cmH_2_O	8	(5.0–8.0)	8	(5.0–8.0)	6.5	(5.0–9.5)	0.641
Driving pressure, cmH_2_O	14	(11.5–15.3)	14	(10.0–15.3)	13.5	(12.0–17.3)	0.486
Thiamine administration	26	(76.5)	20	(76.9)	6	(75)	1
*Blood gas analysis (at the time of intubation)*							
pH	7.35	(7.275–7.424)	7.348	(7.244–7.401)	7.359	(7.299–7.440)	0.73
PaO_2_, mmHg	91.2	(75.3–151.0)	91.2	(78.7–159.3)	96.3	(56.9–107.5)	0.598
PaCO_2_, mmHg	43.5	(33.8–57.7)	42.7	(33.8–57.7)	49.8	(29.8–66.3)	0.612
HCO_3_^−^, mmol/L	22.4	(19.2–25.6)	22.2	(19.5–25.1)	27.2	(19.0–34.2)	0.31
Lactate, mmol/L	1.8	(1.0–4.0)	2.3	(1.2–4.0)	1.5	(1.0–9.4)	0.871
Hemoglobin, g/dL	10.9	(9.0–12.5)	11.1	(10.0–12.7)	8.9	(8.5–11.8)	0.167
*Blood gas analysis (after IC measurement)*							
pH	7.372	(7.312–7.402)	7.375	(7.306–7.404)	7.356	(7.335–7.397)	0.67
PaO_2_, mmHg	94.3	(74.0–116.3)	104	(78.3–120.3	73.5	(67.4–89.9)	0.009
PaCO_2_, mmHg	40.1	(34.5–47.0)	39.5	(34.5–43.3)	47.2	(30.6–52.4)	0.383
HCO_3_^−^, mmol/L	23.4	(20.5–24.8)	23.4	(20.5–24.3)	25	(16.0–31.2)	0.626
Lactate, mmol/L	1.7	(1.1–2.6)	1.5	(1.1–2.5)	2	(1.3–2.6)	0.29
Hemoglobin, g/dL	9.7	(8.8–11.2)	10.4	(9.1–11.3)	9.1	(8.3–10.3)	0.161
Lactate %change, %/h	− 0.98	(− 2.61 to 2.29)	− 1.6	(− 2.82 to 1.74)	2.64	(− 0.94 to 9.40)	0.04
*Vital sign (initial during IC measurement)*							
Heart rate, beat/minute	90.1	(71.2–103.1)	91.3	(69.6–103.4)	89.7	(73.5–95.7)	0.839
Mean arterial pressure, mmHg	76.7	(64.9–88.9)	75.7	(60.5–88.9)	83.6	(66.6–91.5)	0.361
Body temperature, °C	37.2	(36.8–38.0)	37.6	(36.7–38.2)	37.2	(36.8–37.2)	0.3
*Vital sign (last during IC measurement)*							
Heart rate, beat/minute	91.7	(73.6–101.8)	87.4	(67.0–102.7)	91.8	(75.3–96.7)	0.935
Mean arterial pressure, mmHg	80.7	(68.7–91.7)	80.7	(69.2–94.3)	80.7	(62.4–89.2)	0.503
Body temperature, °C	37.3	(36.6–37.7)	37.5	(36.7–38.0)	36.8	(36.3–37.3)	0.074

Summary statistics are reported as No. (%) or medians (interquartile range)

APACHE II, Acute Physiology and Chronic Health Evaluation II; BMI, body mass index; CKD, chronic kidney disease; COPD, chronic obstructive pulmonary disease; CRRT, continuous renal replacement therapy; F_I_O_2_, fraction of inspired oxygen; IC, indirect calorimetry; IQR, interquartile range; RASS, Richmond Agitation-Sedation Scale; SOFA, Sequential Organ Failure Assessment

### Relationship between 28-day survival and VCO_2_, VO_2_ and RQ slopes

**Fig. 2:**
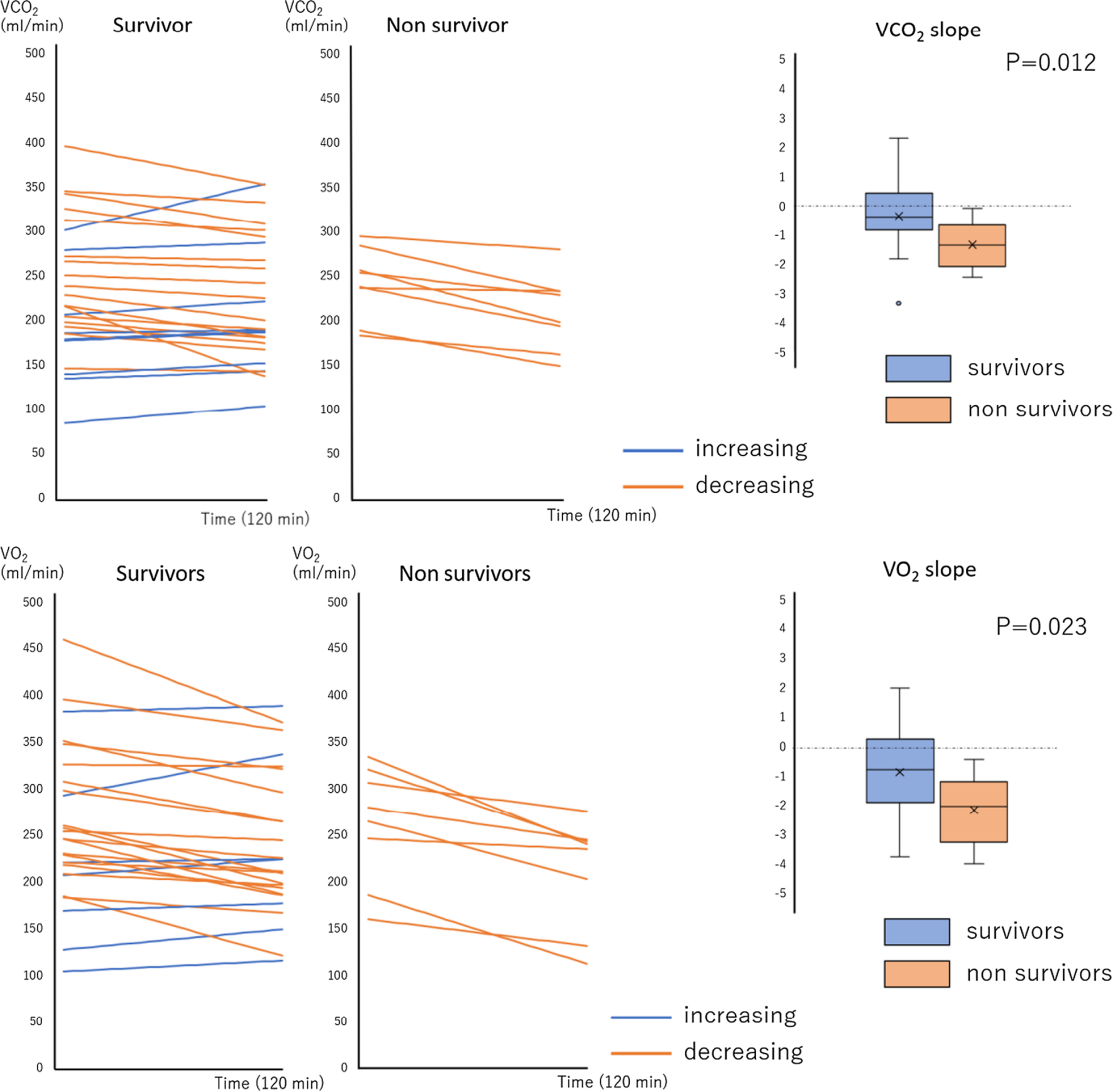
Temporal changes in VCO_2_ and VO_2_. VCO_2_ indicate carbon dioxide production; VO_2_ indicates oxygen extraction. No significant difference in the absolute values of VCO_2_ and VO_2_ was observed between the survivors and non-survivors

Temporal changes of VCO_2_ and VO_2_ are shown in Fig. 2. Median values of VCO_2_ and VO_2_ slopes in survivors and non-survivors showed negative values, indicating temporal reduction of VCO_2_ and VO_2_. Compared with survivors, the absolute values of VCO_2_ and VO_2_ slopes in non-survivors were significantly greater (VCO_2_ slope; − 1.412 vs. − 0.446, *p* = 0.012; VO_2_ slope; − 2.098 vs. − 0.851, *p* = 0.023). However, the slopes of RQ between non-survivors and survivors were not significantly different (RQ slope; 0.001 vs. 0.003, *p* = 0.180) (Additional file 3: Fig. S2). Multiple regression analysis revealed that 28-day mortality was significantly associated with decreases in VCO_2_ slope (adjusted OR (Odds Ratio), 0.349; 95% confidence interval (CI), 0.128–0.953) adjusted of APACHE II score and lactate temporal changes (Table 2). The predictive value of VCO_2_ and VO_2_ slopes for 28-day mortality was evaluated by ROC analysis. We observed significant predictive performance of VCO_2_ and VO_2_ slopes (Additional file 2: Table S2).

**Table 2:** Multivariate logistic regression analysis of 28-day survival

Model1	Variable	OR	95% CI	*P* value
	VCO_2_ slope	0.349	0.128–0.953	0.04
	APACHE II score	1.224	0.962–1.557	0.1
	Lactate %change	1.386	0.995–1.929	0.053

Model2	Variable	OR	95% CI	*P* value
	VCO_2_ slope	0.602	0.289–1.255	0.176
	APACHE II score	1.166	0.947–1.436	0.148
	Lactate %change	1.282	0.990–1.660	0.059

APACHE II, Acute Physiology and Chronic Health Evaluation II; CI, confidence interval; OR, odds ratio; VCO_2_, carbon dioxide production; VO_2_, oxygen extraction

### Combination of VCO_2_ and VO_2_ slopes with lactate temporal change

**Fig. 3:**
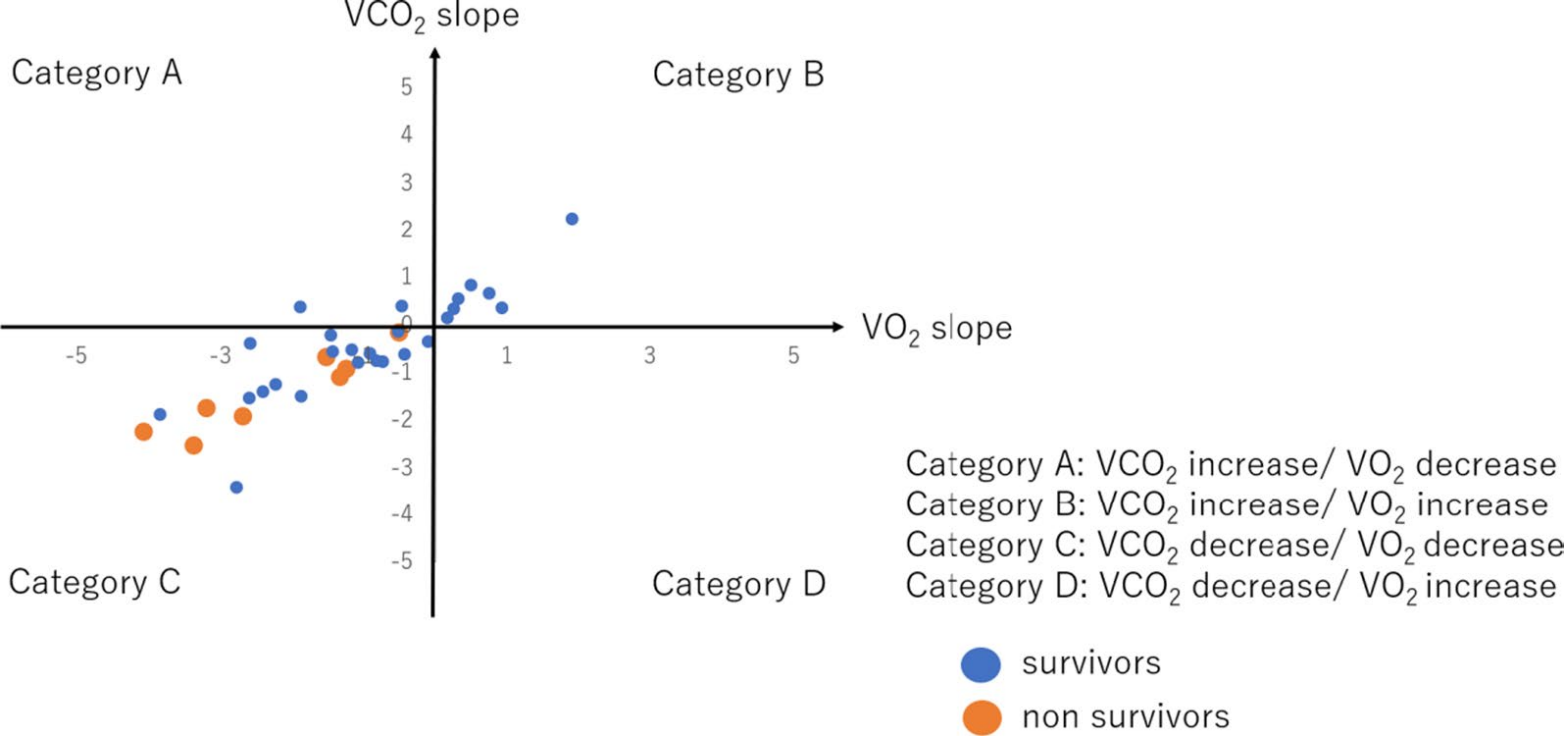
Two-dimensional analysis of VCO_2_ and VO_2_. VCO_2_ indicates carbon dioxide production; VO_2_ indicates oxygen extraction

**Fig. 4:**
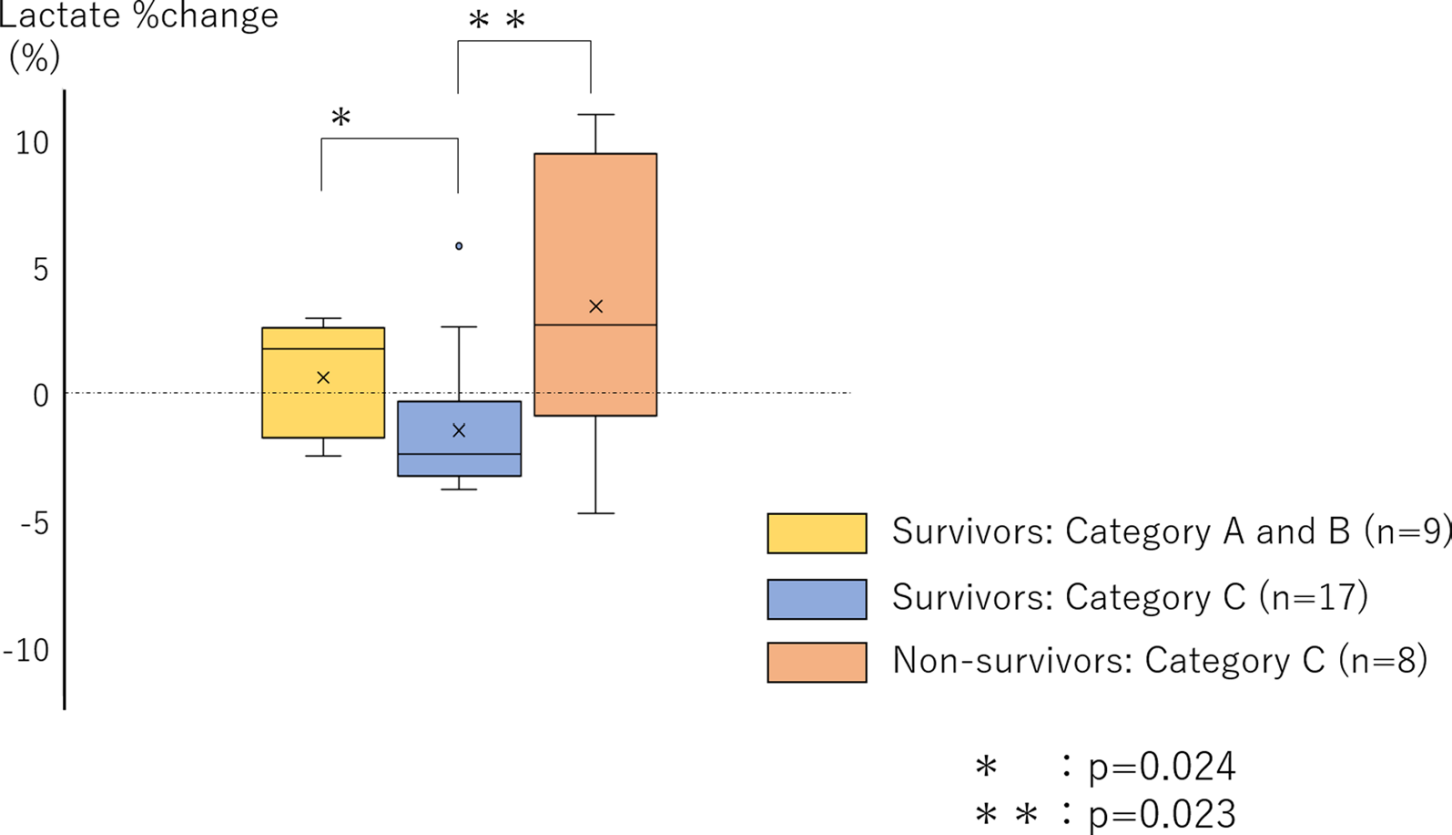
Difference in lactate temporal changes among the three groups

The patients were categorized into four groups according to combinations of VCO_2_ and VO_2_ slopes (Fig. 3). All non-survivors and 17 of the 26 survivors showed negative VCO_2_ and VO_2_ slopes (category C). No patients were classified into category D. Among the patients in category C, lactate temporal changes in the non-survivors were significantly higher than those in the survivors (i.e., lower lactate clearance) (2.6 vs. − 2.4, respectively) (*p* = 0.023) (Fig. 4). Among the survivors, those in category C had significantly lower lactate temporal changes than those in categories A and B (− 2.4 vs. 1.7, respectively) (*p* = 0.024).

## Discussion

### Principal findings

Among the intubated patients with sepsis, all the non-survivors showed temporal declines in both VCO_2_ and VO_2_. Temporal changes in blood lactate levels provided additional information for discriminating non-survivors from survivors when patients showed temporal decreases in VCO_2_ and VO_2_. In contrast, survivors with temporal elevations in VCO_2_ or VO_2_ showed significantly lower lactate clearance than other survivors. Taken together, declines in VCO_2_ and VO_2_ and lower lactate clearance seemed to be risk factors for poor outcomes. These findings suggest that temporal changes in VCO_2_ and VO_2_ with lactate clearance will enable us to monitor the severity of dysregulation of carbon dioxide production and oxygen consumption in sepsis and predict the outcome of death.

### Comparison with other studies

Because one of the major aspects of the pathophysiology of sepsis-induced organ dysfunction is failure of carbon dioxide production and oxygen use in cellular mitochondria [11], VCO_2_ or VO_2_ measurements are expected to provide physiological information on the severity of sepsis. Several studies have reported that VCO_2_ or VO_2_ can be measured using IC in patients with sepsis. Hoeyer-Nielsen et al. recently have shown a higher VO_2_ lactate ratio in survivors and a significant difference in temporal VCO_2_ changes between survivors and non-survivors [16]. This study used three parameters (i.e., VCO_2_, VO_2_, and lactate clearance) and found possible additive values by combining these three parameters. These two studies suggested the importance of temporal change in physiological parameters such as VCO_2_ and VO_2_. Evaluation only with absolute values will be hampered by individual differences, which will be frequently observed in severe sepsis patients. Of note, previous studies reported the superiority of temporal changes compared with absolute value of lactate in sepsis patients [8, 24]. Temporal evaluation can overcome the problem of individual variation and may contribute to personalized medicine. It should be noted that indirect calorimetry used in this study enabled continuous monitoring of VCO_2_ and VO_2_.

S_CV_O_2_ and lactate clearance are predictors of mortality in patients with sepsis [5–8]. However, these clinical parameters have several limitations to be considered. In septic conditions, microcirculatory heterogeneity that generates capillary shunting frequently elevates S_CV_O_2_ values [9]. Hyperlactatemia does not necessarily reflect sepsis progression as other diseases, such as liver dysfunction, seizure, and diabetic ketoacidosis, could cause hyperlactatemia [10].

### Study strength

**Fig. 5:**
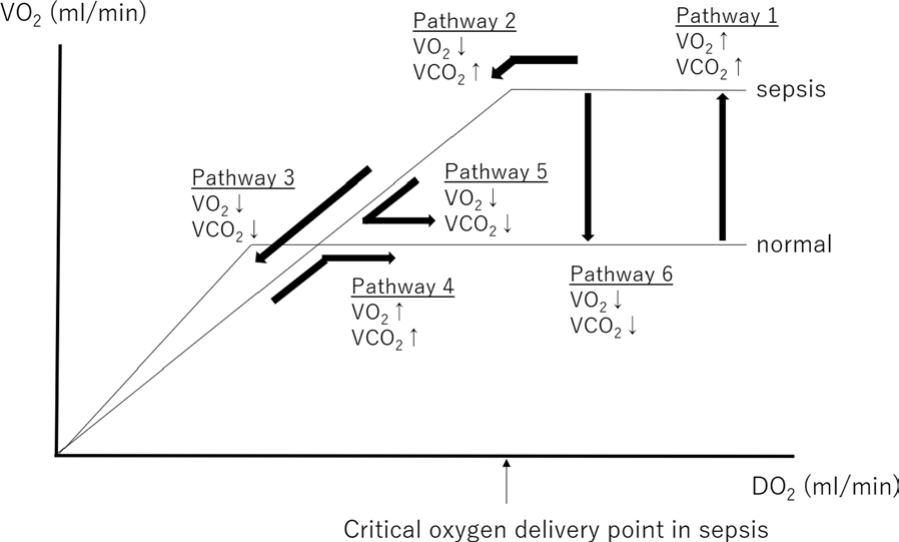
Changes in VCO_2_ and VO_2_ in sepsis. VCO_2_ indicates carbon dioxide production; VO_2_ indicates oxygen extraction; DO_2_ indicates oxygen delivery

Figure 5 shows a speculation regarding temporal changes in VCO_2_ and VO_2_ along with DO_2_ in patients with sepsis. VO_2_ reaches a plateau at a higher level of DO_2_ (critical oxygen delivery point) [25]. Due to cytopathic hypoxia, sepsis reduces the slope of the VO_2_/DO_2_ ratio. Patients with sepsis require high VO_2_ [26]. This change will also increase VCO_2_ (Pathway 1 in Fig. 5). When DO_2_ is below the critical oxygen delivery point, VO_2_ will also decrease (Pathway 2 in Fig. 5). Further reduction in DO_2_ will induce anaerobic metabolism, and VCO_2_ will decrease (Pathway 3 in Fig. 5). When some therapeutic interventions successfully increase VCO_2_, VO_2_ will increase (Pathway 4 in Fig. 5). A decrease in VCO_2_ will be observed during the recovery phase in which VO_2_ is decreased (Pathways 5 and 6 in Fig. 5). All non-survivors and some survivors in this study showed a dual reduction in VCO_2_ and VO_2_ (Pathways 3, 5, and 6), and we assumed that non-survivors and survivors with VCO_2_ and VO_2_ reductions correspond to Pathways 3 and 5 or 6, respectively. Continuous monitoring of VCO_2_ and VO_2_ could provide valuable information for determining management of sepsis and be expected to be a useful indicator for therapeutic interventions to sepsis patients. However, evaluating temporal changes in VCO_2_ and VO_2_ alone could not discriminate non-survivors from survivors. Additional information, especially lactate temporal changes, will provide a better prediction performance. In contrast, patients with increased VCO_2_ or VO_2_ had a good prognosis despite lactate elevation. Although this speculation must be confirmed with a larger population, the combination of VCO_2_ and VO_2_ measurement using IC with lactate temporal changes may be expected to be a good predictor of prognosis in sepsis.

### Limitations

This study has several limitations. First, we focused on the temporal changes in VCO_2_ and VO_2_ values instead of their absolute values for eliminating individual variations among patients and the measurement procedure. However, obtaining the temporal data and evaluating relative changes are time-consuming. Second, the obtained results in this study should be confirmed using other IC models. No changes in mechanical ventilation for F_I_O_2_ were required because a change in supplementary oxygen during IC measurement can lead to inaccurate data [27]. This will be a barrier for clinical application. Third, all data were obtained, while no nutritional support was provided. Additional VCO_2_ production by nutrition should be considered in the later phase of ICU stay. Fourth, severe metabolic acidosis induces excess carbon dioxide excretion from the lungs as compensation. Other factors that may affect changes in VCO_2_ and VO_2_ during IC measurement, such as sedation level, body temperature, cardiac function including cardiac output, hemoglobin, and S_CV_O_2_, should also be evaluated. Fifth, factors affecting lactate levels, such as hepatic function and lack of thiamine, were not examined. Finally, the sample size was small because this is a single-center cohort study. We used only APACHE II score and temporal lactate changes as variables for multivariate analysis to avoid overfitting. Further studies involving larger cohorts and analysis considering sufficient possible confounding factors are necessary to confirm our conclusions. Because the results of this study are preliminary, our approach cannot be immediately clinically applied. Clinical application will be possible when the profound technological improvement on IC will allow us to monitor VO_2_ and VCO_2_ more easily as end-tidal carbon dioxide monitoring, which is widely used in mechanical ventilated patients.

## Conclusion

Among mechanically ventilated patients with sepsis, non-survivors showed temporal decreases in VCO_2_ and VO_2_ with lactate elevation. Monitoring changes in VCO_2_ and VO_2_ along with lactate changes will provide information on tissue metabolism in sepsis and may be useful in predicting the prognosis of patients with sepsis.

## Supplementary Information

**Additional file 1. Figure S1.** Data processing method.

**Additional file 2. Table S1.** Average values of VCO_2_, VO_2_, RQ, and REE during 2 h IC measurement. **Table S2.** ROC analysis for 28-day survival.

**Additional file 3. Figure S2.** Temporal change of RQ.

## Data Availability

The datasets used and/or analyzed in this study are available from the corresponding author on reasonable request.

## Abbreviations

APACHE: Acute Physiology and Chronic Health Evaluation
AUC: Area under the curve
CI: Confidence interval
DO_2_: Oxygen delivery
ECMO: Extracorporeal membrane oxygenation
F_I_O_2_: Fraction of inspired oxygen
IC: Indirect calorimetry
ICUs: Intensive care units
OR: Odds ratio
RASS: Richmond Agitation-Sedation Scale
ROC: Receiver operating characteristic
RQ: Respiratory quotient
S_CV_O_2_: Central venous oxygen saturation
SOFA: Sequential Organ Failure Assessment
VCO_2_: Carbon dioxide production
VO_2_: Oxygen extraction
