# Supplementary figures and images for: Changes in carbon dioxide production and oxygen uptake evaluated using indirect calorimetry in mechanically ventilated patients with sepsis
Source: Crit Care. 2021 Dec 4;25:416. doi: 10.1186/s13054-021-03830-z (PMC8645073; doi:10.1186/s13054-021-03830-z)

## Slide 1
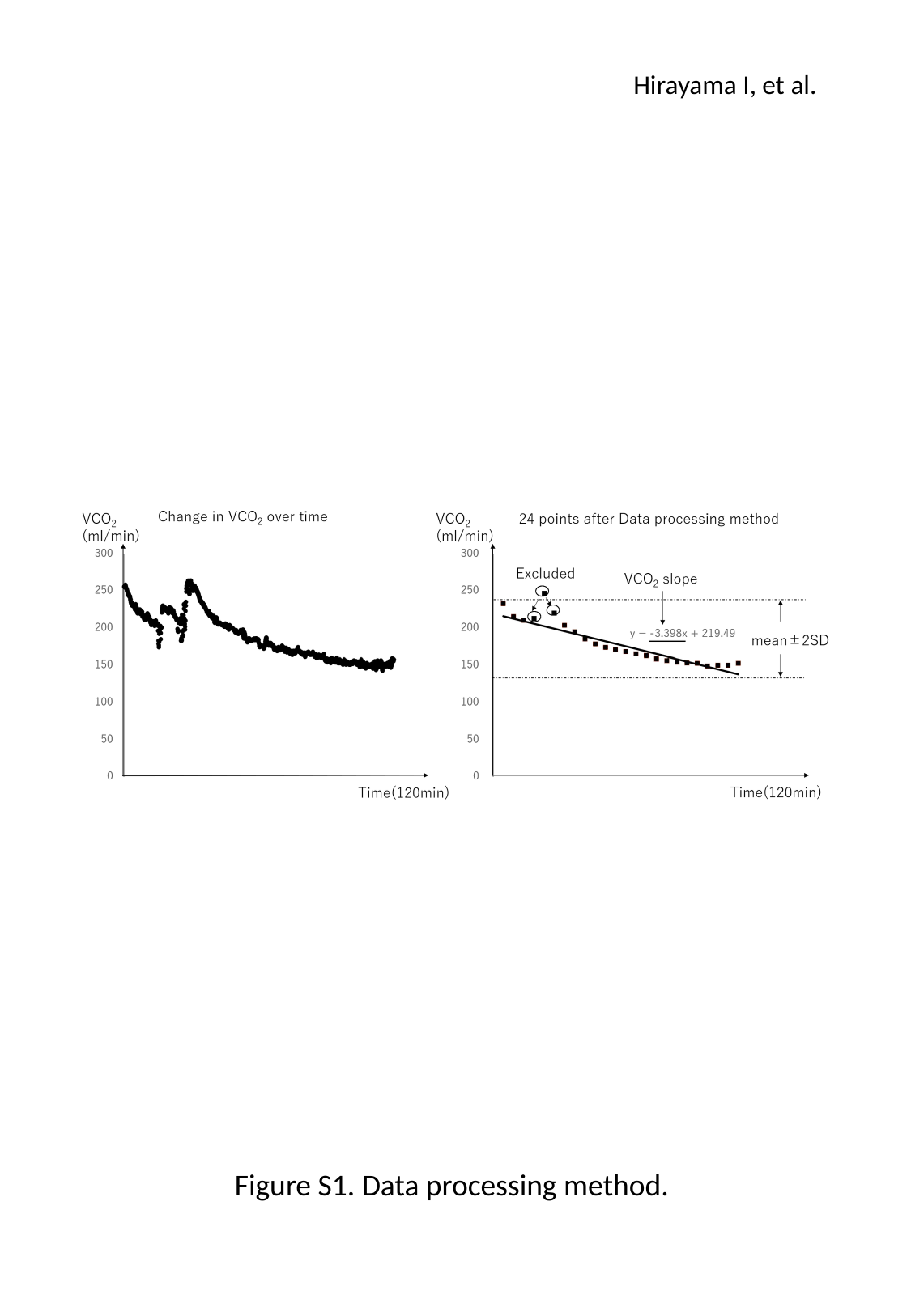

Hirayama I, et al.
Figure S1. Data processing method.

Supplement: Supplementary file 1 — Additional file 1. Figure S1. Data processing method. [file 13054_2021_3830_MOESM1_ESM.pptx]

## Slide 1
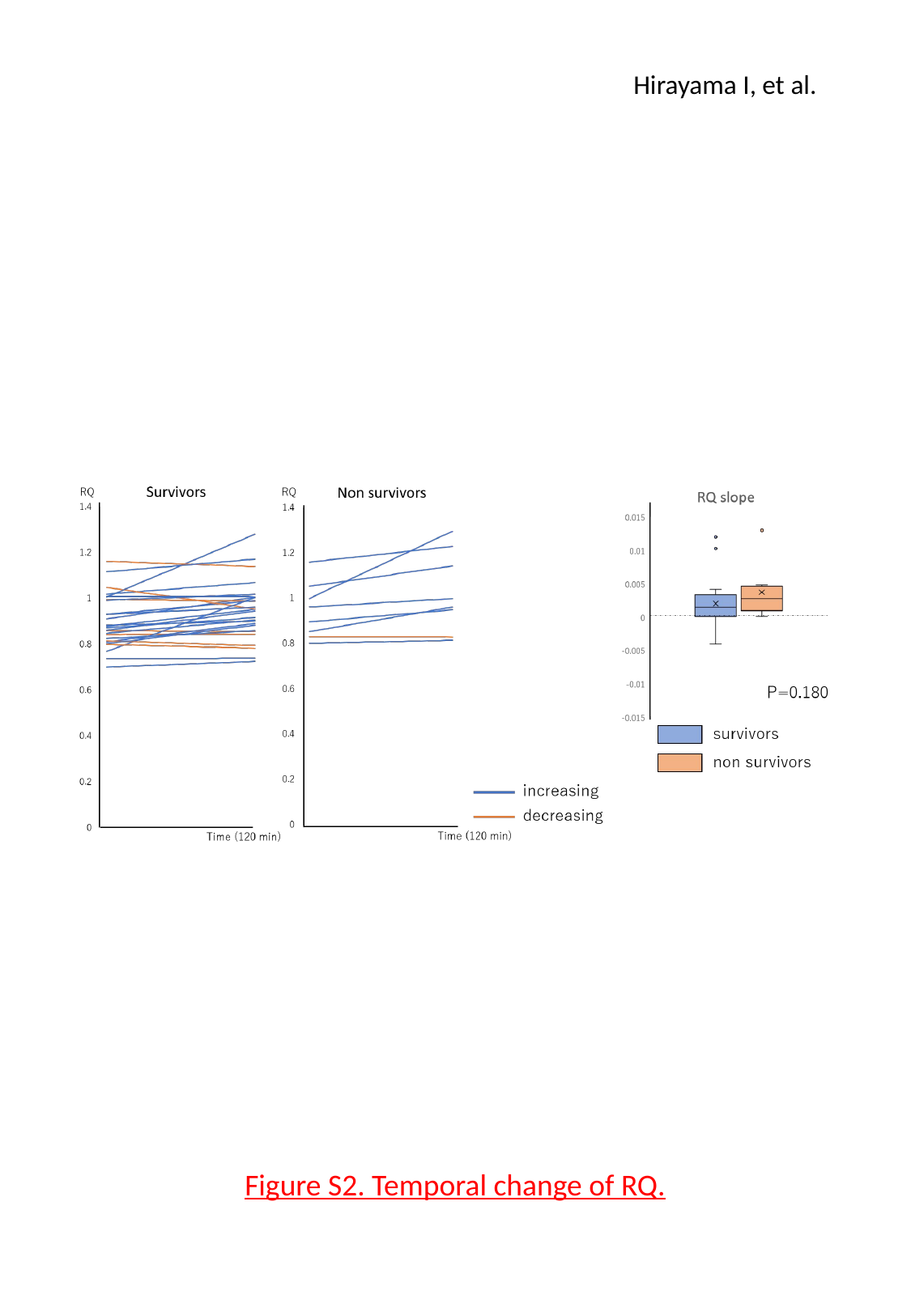

Hirayama I, et al.
Figure S2. Temporal change of RQ.

Supplement: Supplementary file 3 — Additional file 3. Figure S2. Temporal change of RQ. [file 13054_2021_3830_MOESM3_ESM.pptx]
