# Supplementary material for: Changes in carbon dioxide production and oxygen uptake evaluated using indirect calorimetry in mechanically ventilated patients with sepsis
Source: Crit Care. 2021 Dec 4;25:416. doi: 10.1186/s13054-021-03830-z (PMC8645073; doi:10.1186/s13054-021-03830-z)
Supplement: Supplementary file 2 — Additional file 2. Table S1. Average values of VCO2, VO2, RQ, and REE during 2 h IC measurement. Table S2. ROC analysis for 28-day survival. [file 13054_2021_3830_MOESM2_ESM.docx]

Table S1. Average values of VCO_2_, VO_2_, RQ, and REE during 2 h IC measurement.

|  | Patient | VCO_2_  ml/min | VO_2_  ml/min | RQ | REE  kcal |
| --- | --- | --- | --- | --- | --- |
| non survivor | 1 | 219.6 | 239.1 | 0.92 | 1707.5 |
| non survivor | 2 | 232.0 | 288.1 | 0.81 | 2005.1 |
| non survivor | 3 | 256.9 | 283.4 | 0.91 | 2018.3 |
| non survivor | 4 | 288.2 | 262.7 | 1.10 | 1951.4 |
| non survivor | 5 | 239.8 | 289.0 | 0.83 | 2023.1 |
| non survivor | 6 | 235.9 | 241.3 | 0.98 | 1746.3 |
| non survivor | 7 | 172.7 | 145.4 | 1.20 | 1101.0 |
| non survivor | 8 | 169.1 | 148.9 | 1.15 | 1115.1 |
| Survivor | 9 | 198.0 | 250.4 | 0.79 | 1737.3 |
| Survivor | 10 | 331.5 | 318.4 | 1.05 | 2337.9 |
| Survivor | 11 | 185.0 | 207.1 | 0.89 | 1470.8 |
| Survivor | 12 | 270.3 | 281.4 | 0.96 | 2028.7 |
| Survivor | 13 | 216.2 | 218.2 | 0.99 | 1583.4 |
| Survivor | 14 | 189.0 | 220.2 | 0.86 | 1551.2 |
| Survivor | 15 | 307.7 | 319.3 | 0.97 | 2303.3 |
| Survivor | 16 | 214.1 | 233.2 | 0.92 | 1665.3 |
| Survivor | 17 | 284.0 | 386.4 | 0.74 | 2646.2 |
| Survivor | 18 | 372.0 | 409.0 | 0.91 | 2915.2 |
| Survivor | 19 | 188.5 | 223.6 | 0.85 | 1569.9 |
| Survivor | 20 | 97.1 | 112.4 | 0.86 | 792.6 |
| Survivor | 21 | 139.8 | 140.2 | 1.00 | 1019.2 |
| Survivor | 22 | 185.1 | 203.4 | 0.91 | 1449.9 |
| Survivor | 23 | 230.5 | 233.1 | 1.01 | 1691.2 |
| Survivor | 24 | 339.2 | 378.1 | 0.90 | 2687.3 |
| Survivor | 25 | 176.1 | 175.1 | 1.01 | 1275.0 |
| Survivor | 26 | 246.2 | 211.9 | 1.17 | 1596.0 |
| Survivor | 27 | 262.1 | 325.7 | 0.81 | 2266.5 |
| Survivor | 28 | 173.4 | 150.9 | 1.15 | 1133.2 |
| Survivor | 29 | 200.8 | 230.8 | 0.88 | 1630.4 |
| Survivor | 30 | 308.5 | 336.9 | 0.95 | 2404.5 |
| Survivor | 31 | 182.5 | 216.6 | 0.84 | 1520.5 |
| Survivor | 32 | 328.1 | 288.9 | 1.14 | 2163.5 |
| Survivor | 33 | 144.8 | 202.0 | 0.72 | 1377.6 |
| Survivor | 34 | 144.7 | 172.8 | 0.84 | 1211.4 |

IC, indirect calorimetry; REE, resting energy expenditure; RQ, respiratory quotient; VCO_2_, carbon dioxide production; VO_2_, oxygen extraction.

Table S2. ROC analysis for 28-day survival.

|  | AUC | 95%CI | cut off value | sensitivity | specificity |
| --- | --- | --- | --- | --- | --- |
| VCO_2_ slope | 0.798 | 0.577-0.920 | -0.656 | 0.875 | 0.654 |
| VO_2_ slope | 0.769 | 0.542-0.904 | -1.228 | 0.875 | 0.615 |
| APACHE II score | 0.736 | 0.511-0.881 | 17.0 | 0.875 | 0.577 |
| Lactate %change | 0.743 | 0.439-0.914 | 3.5 | 0.500 | 0.962 |

APACHE II, Acute Physiology and Chronic Health Evaluation II; AUC, area under the curve; CI, confidence interval; VCO_2_, carbon dioxide production; VO_2_, oxygen extraction.
